# Effectiveness of maturity of *Rubus occidentalis* on hyperalgesia induced by acidic saline injection in rats

**DOI:** 10.1186/s12906-021-03491-z

**Published:** 2022-01-11

**Authors:** Geun Joo Choi, Hyun Kang, Oh Haeng Lee, Eun Jin Ahn, Fletcher A. White, Ye Jin Cho, Chong Wha Baek, Yong Hun Jung, Ji Wung Kwon

**Affiliations:** 1 Department of Anesthesiology and Pain Medicine, Chung-Ang University College of Medicine, 84 Heukseok-ro, Dongjak-gu, Seoul, 06911 Republic of Korea; 2 Department of Anesthesia, Indiana University School of Medicine, Indianapolis, IN 46202 USA; 3 Stark Neurosciences Research Institute, Indiana University School of Medicine, Indianapolis, IN 46202 USA; 4 Gochang Berry & Bio Food Research Institute, Gochang-gun, Jeollabuk-do South Korea

**Keywords:** Analgesics: Black raspberry, Chronic pain, Hyperalgesia, *Rubus occidentalis*

## Abstract

**Background:**

*Rubus occidentalis*, also known as black raspberry, contains several bioactive components that vary depending on the maturity of the fruit. The goal of this study was to evaluate the efficacy of immature *Rubus occidentalis* extract(iROE) on acid-induced hyperalgesia, investigate the mechanism involved, and compare the antihyperalgesic effect of immature and mature ROEs.

**Methods:**

In adult male Sprague-Dawley rats, chronic muscle pain was induced via two injections of acidic saline into one gastrocnemius muscle. To evaluate the dose response, the rats were injected intraperitoneally with 0.9% saline or iROE (10, 30, 100, or 300 mg/kg) following hyperalgesia development. To evaluate the mechanism underlying iROE-induced analgesia, the rats were injected intraperitoneally with saline, yohimbine 2 mg/kg, dexmedetomidine 50 μg/kg, prazosin 1 mg/kg, atropine 5 mg/kg, mecamylamine 1 mg/kg, or naloxone 5 mg/kg 24 h after hyperalgesia development, followed by iROE 300 mg/kg administration. To compare immature versus mature ROE, the rats were injected with mature ROE 300 mg/kg and immature ROE 300 mg/kg after hyperalgesia development. For all experiments, the mechanical withdrawal threshold(MWT) was evaluated using von Frey filaments before the first acidic saline injection, 24 h after the second injection, and at various time points after drug administration. Data were analysed using multivariate analysis of variance(MANOVA) and the linear mixed-effects model(LMEM). We compared the MWT at each time point using analysis of variance with the Bonferroni correction.

**Results:**

The iROE 300 mg/kg injection resulted in a significant increase in MWT compared with the control, iROE 30 mg/kg, and iROE 100 mg/kg injections at ipsilateral and contralateral sites. The iROE injection together with yohimbine, mecamylamine, or naloxone significantly decreased the MWT compared with iROE alone, whereas ROE together with dexmedetomidine significantly increased the MWT. According to MANOVA, the effects of immature and mature ROEs were not significantly different; however, the LMEM presented a significant difference between the two groups.

**Conclusions:**

Immature *R. occidentalis* showed antihyperalgesic activity against acid-induced chronic muscle pain, which may be mediated by the α_2_-adrenergic, nicotinic cholinergic, and opioid receptors. The iROE displayed superior tendency regarding analgesic effect compared to mature ROE.

**Supplementary Information:**

The online version contains supplementary material available at 10.1186/s12906-021-03491-z.

## Background

Chronic musculoskeletal pain has a considerable influence on physical, emotional, psychological, and social aspects with only few effective therapeutic strategies available for pain control [1]. Currently, multimodal therapeutic approaches using various medications and techniques are recommended for chronic pain control; however, this condition remains difficult to treat. The pharmacological regimen for pain management tends to emphasise opioid sparing. Moreover, pharmacological strategies for pain control should be safe to enable their use over a long duration. In this regard, recently, there is a growing interest in therapeutic approaches based on food for the management of chronic pain. Indeed, several investigations have suggested the health benefits of functional foods or nutraceuticals and their potential for relieving chronic pain [2].

*Rubus occidentalis*, also known as black raspberry, is being recognised for its anti-inflammatory, antinociceptive, and antioxidant properties [3, 4]. These properties of *R. occidentalis* are attributed to its content of anthocyanins, ellagic acid, and other phenolic compounds, the bioactivities of which vary depending on the maturity of the fruit [3, 5, 6]. In a study on pre-matured black raspberry, immature *R. occidentalis* appeared to produce a high content of phenolic compound, flavonoids, and vitamin C, implying the potential to be used as a functional food or medicinal substance derived from nature [5].

Previously, we investigated the effect of mature *R. occidentalis* on chronic pain model in rats [7] and there has been no study regarding the effect of immature *R. occidentalis* on chronic pain and associated mechanism yet. Therefore, we hypothesise that the immature form of *R. occidentalis* will attenuate chronic musculoskeletal pain in current study. To identify the relationship between chronic musculoskeletal pain and immature *R. occidentalis*, we developed a rat model with hyperalgesia induced by repeated intramuscular injection of acidic saline; immature *R. occidentalis* extract (iROE) was administered intraperitoneally. The primary outcome was to assess the analgesic property of iROE. The secondary outcome was to investigate the potential mechanism underlying its analgesic activity. Additionally, we compared the analgesic effect between immature and mature *R. occidentalis*.

## Methods

This randomised controlled experimental study was performed in accordance with the National Institutes of Health Guide for the Care and Use of Laboratory Animals and described according to the Animal Research: Reporting In Vivo Experiments (ARRIVE) guidelines [8, 9].

### Preparation of the immature or mature *R. occidentalis* extract

The iROE and mature *R. occidentalis* extract (mROE) were provided by the Gochang Black Raspberry Research Institute of South Korea.

In June 2016, immature fruit with green colour was harvested within post-bloom 28 days and mature fruit with dark red colour was harvested after post-bloom 38 days from the Gochang (Jeollabuk-Do) area of South Korea. Both immature and mature *R. occidentalis* were authenticated by Dr. Ji Wung Kwon, a research director of Gochang Black Raspberry Research Institute, where voucher specimens of the two were deposited (specimen voucher number: GBRI-17, 18).

The following two extract of *R. occidentalis* fruit: iROE and mROE were prepared. In brief, immature fruit (1 kg) and mature fruit (1 kg) were pulverized and extracted twice with 50% ethanol (10 times weight of each fruit) at 80 °C for 2 h by using a reflux condenser. The extract was filtered and concentrated, and the concentrated was lyophilised in a freezer-dryer and stored at − 20 °C before use. The extract yields were 17.2% of immature fruit and 7.5% of mature fruit, respectively. Since the ethanol was effective and stable solvent for plant extraction in many studies on the *Rubus* species, 50% ethanol for fruit extraction was used. All procedures were conducted under sterilized condition and stored iROE and mROE were used just before intraperitoneal administration.

### Study animals

The experiment was performed in the Animal Research Laboratory of Chung-Ang University and was approved by the Institutional Animal Care and Use Committee at Chung-Ang University (no. 2017–00107). Adult male Sprague-Dawley rats (250–300 g; Coretec, Seoul, Korea) were housed individually in cages in a temperature-controlled room (22 °C) and fed a standard laboratory diet and tap water ad libitum. They were maintained under a 12-h light/dark cycle (lights on from 8:00 a.m. to 8:00 p.m.) and acclimated to the housing facilities for 1 week before the experimental procedures. Females were not included because hormonal fluctuations may have affected the pain threshold [10]. Rats with any abnormalities were excluded. All animals were euthanized by placing rats in the transparent induction chamber and carbon dioxide (CO_2_) inhalation after completing the experiment [11]. Specifically, we used a compressed CO_2_ gas cylinder with a pressure-reducing regulator and a calibrated flow meter. We placed rats in the chamber without pre-charging the chamber and introduced 100% CO_2_ at flow rate of 3 to 7 l per minute, equivalent to a fill rate of 30 to 70% of the 10-l chamber volume per minute.

### Induction of hyperalgesia in the muscle

All the experiments were performed between 8 a.m. and 1 p.m. to avoid diurnal variation and under sterile conditions by an investigator who was blinded to the group allocation of the individual rats. Hyperalgesia was induced in the muscle as described previously [12], with minor modifications on injection interval in the reported technique. Briefly, rats were anaesthetised with 1–4% isoflurane in 100% oxygen and injected with 100 μL of pH 4.0 preservative-free sterile saline into a lateral gastrocnemius muscle on day 0 and again on day 3.

### Group allocation and blinding

To evaluate the antinociceptive effect of iROE and elucidate the mechanism underlying the iROE-induced analgesia, the rats were randomly divided into groups according to the respective experiment. Random assignment was based on a table generated using the PASS software, version 11 (NCSS, Kaysville, UT, USA), applying Wei’s urn model. The randomisation code was generated by a statistician who was not otherwise involved in the study. For allocation concealment, another investigator who was not involved in this study prepared syringes containing the study drugs for the experiments. The study drugs were dissolved in normal saline, and the intraperitoneal (IP) injection volume was 2 mL. The syringes were covered with an opaque tape and numbered sequentially according to a randomised list of experiments. The prepared syringes were delivered to a researcher in charge of surgery. This researcher, who was blinded to the group assignment, participated only in the peritoneal injection.

### Experiment 1: Evaluation of the antinociceptive effect of iROE: Dose-response test

The purpose of Experiment 1 was to evaluate the antinociceptive effect of iROE on the mechanical hyperalgesia induced by repeated intramuscular injections of acidic saline. Fifty rats were randomly assigned to 1 of 5 groups of 10 rats (groups administered control or 10, 30, 100, or 300 mg/kg iROE). Various doses of iROE or normal saline was injected intraperitoneally 24 h after the second injection of acidic saline. The doses of ROE were referred on the amount used in our previous experimental study [7] and fixed based on the logarithmic increase.

### Experiment 2: Elucidation of the mechanism mediating iROE-induced analgesia

The purpose of Experiment 2 was to examine whether the antinociceptive effect of iROE was mediated via α (1 and 2)-adrenergic, cholinergic (nicotinic and muscarinic), and/or opioid receptors. Seventy rats were randomly assigned to 1 of 7 groups of 10 rats, which were injected with either normal saline (iROE 300 mg/kg alone) or yohimbine 2 mg/kg, dexmedetomidine 50 μg/kg, prazosin 1 mg/kg, atropine 5 mg/kg, mecamylamine 1 mg/kg, or naloxone 5 mg/kg, 24 h after the development of hyperalgesia. Ten minutes later, 300 mg/kg iROE was injected intraperitoneally. Previous research [13–16] supported the use of drugs to elucidate the possible involvement of those receptor systems. Sigma Aldrich provided us with the study drugs (U.S.A.).

### Experiment 3: Comparison of the antinociceptive effect of iROE and mROE

The purpose of Experiment 3 was to evaluate the difference in the antinociceptive effect of iROE and mROE on the mechanical hyperalgesia induced by repeated intramuscular injections of acidic saline. Two groups of 10 rats each were administered mROE 300 mg/kg and iROE 300 mg/kg, respectively. The dose of immature and mature ROEs was determined based on the results from dose response test from present and previous studies.

### Experiment 4: Comparison of the antinociceptive effect of iROE versus the positive control

The purpose of Experiment 4 was to assess the validity of the present study. The group receiving the peak effective dosage of iROE (300 mg/kg) for antinociception was compared to a positive-control group receiving intraperitoneal ketolorac (30 mg/kg).

### Experiment 5: Assessment of motor impairment

To identify the effect of iROE on motor function or the sedative effect of iROE, we used an accelerating Rota-rod treadmill (Jeung Do Bio & Plant Co., Ltd., Seoul, Korea). This test is useful for determining motor impairment caused by pharmacological agents such as central nervous system depressants. Eighteen rats were randomly assigned to one of three groups of six rats: iROE 300 mg/kg group, mROE 300 mg/kg group, and control group. To adapt the rats to the treadmill test, 2 sets of training trials were performed 2 days and 1 day before the first injection. The rats were injected intraperitoneally with 300 mg/kg iROE, mROE, or normal saline 24 h after the second injection of acidic saline. Two hours after the injection of iROE, mROE, or normal saline, the Rota-rod test was performed. Specifically, the rats were placed on the Rota-rod running at a speed gradually increasing from 1 to 18 rotations per minute (rpm) for 120 s and maintained for another 30 s at 18 rpm [12]. The time point at which the rats fell off the Rota-rod was noted.

### Behavioural measurements

For experiments 1, 2, 3, and 4, individual rats were placed on an elevated plastic mesh floor (8 × 8 mm perforations) under an overturned clear plastic cage (21 × 27 × 15 cm) and allowed to acclimate for 15 min. The rats were then evaluated to determine their withdrawal thresholds to mechanical stimuli using von Frey filaments (Stoelting Co., IL, USA). The filaments were applied vertically to the plantar aspect of the hind paw by administering sufficient pressure to gently bend the filament. Filaments with bending forces of 4, 9, 20, 59, 78, 98, 147, and 254 mN were progressively applied until the hind paw was withdrawn or a bending force of 254 mN (the cut-off value) was reached. Each filament was applied three times at intervals of 3 min. The lowest bending force that caused paw withdrawal after the application of the filament was considered the mechanical withdrawal threshold (MWT) of the hind paw. The full lifting of the plantar surface off the mesh floor was considered a positive withdrawal response and partial lifting, walking, hunching, stretching, or licking was not included. After a response was observed, filaments with higher and lower bending forces were utilised to confirm the MWT. The MWT assessment was performed by a well-trained investigator who was unaware of the group allocation.

The MWT was assessed according to the following schedule: before the first injection (BI); 24 h after the second injection (AI); and 15 min, 30 min, 45 min, 60 min, 80 min, 100 min, 120 min, 24 h, 48 h, and 7 days after the injection of the test drugs. The area under the curve (AUC) values were obtained for 120 min (from AI to 120 min after the injection of the test drugs).

### Statistical analysis

Data were analysed using analysis of variance (ANOVA), one-way Wilk’s lambda multivariate analysis of variance (MANOVA) followed by post hoc t-test or univariate ANOVA with the Bonferroni correction and linear mixed-effects model. Individual measurements are expressed as the means ± standard errors in the figures and were analysed using SPSS 23.0 (IBM Corp., Armonk, NY, USA). A *P* value of 0.05 or less was considered statistically significant. A detailed description of the statistical analysis is provided in the supplementary file.

## Results

### Study animals

All of the rats completed the present study. Throughout the experimental period, the rats remained well-groomed and appeared to ingest a normal amount of food and water. All rats completed the study and no complications were observed.

### Evaluation of the antinociceptive effect of iROE: Dose-response test

**Fig. 1:**
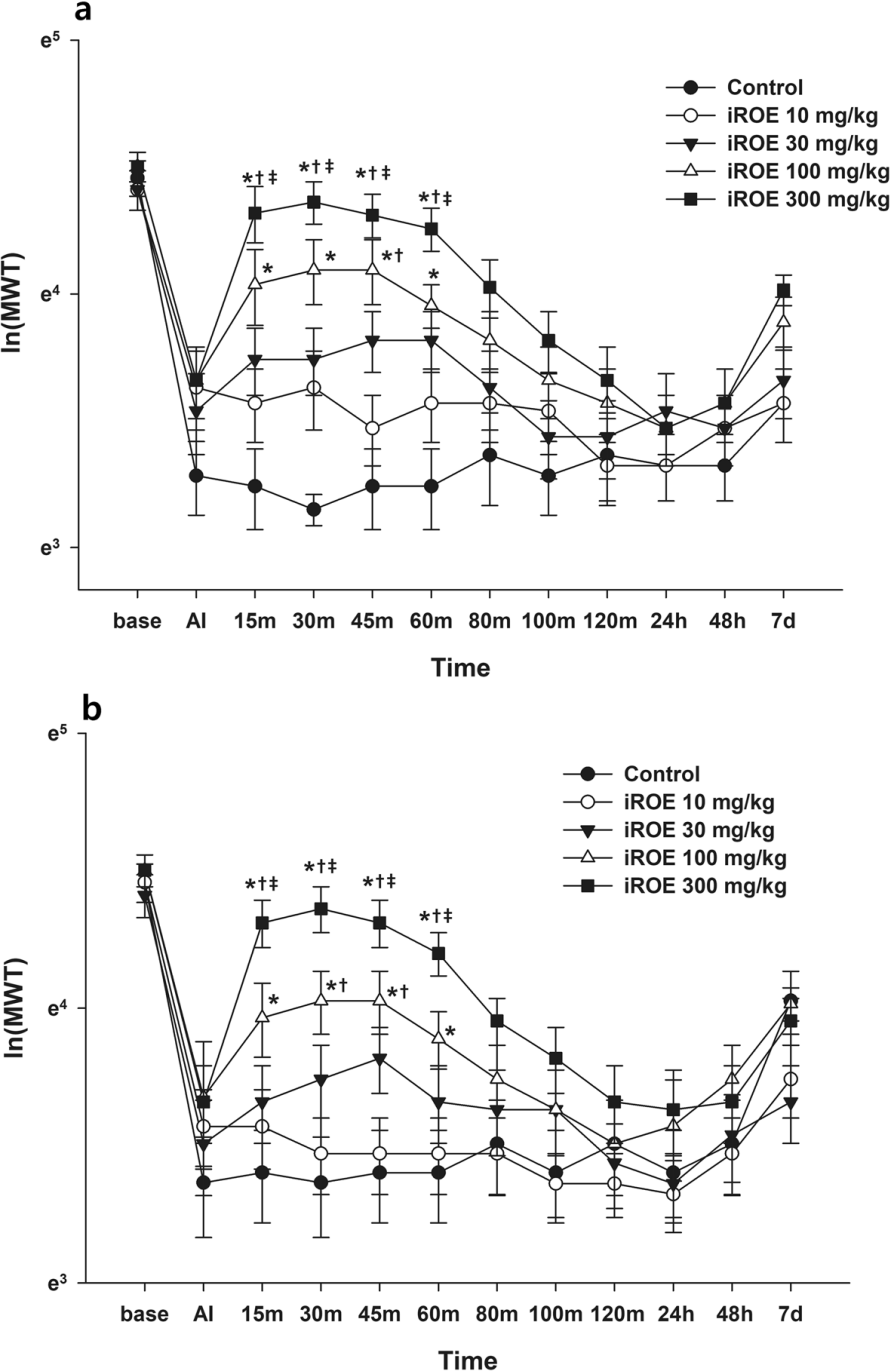
Antinociceptive effect of immature *Rubus occidentalis* extract (iROE). **a** ipsilateral site, **b** contralateral site. AI: immediately after injection. For statistical analysis, one-way Wilk’s lambda multivariate analysis of variance followed by post hoc univariate ANOVA with the Bonferroni correction and linear mixed-effects model was used. *P < 0.05 compared with the control group, †*P* < 0.05 compared with the iROE 10 mg/kg group, ‡*P* < 0.05 compared with the iROE 30 mg/kg group

The results of MANOVA at the ipsilateral and contralateral sites showed a statistically significant difference among the groups (F(48, 133.010) = 1.529, *P* = 0.031: Wilk’s lambda = 0.184, partial η^2^ = 0.345 and F(48, 133.010) = 1.491, *P* = 0.039: Wilk’s lambda = 0.190, partial η^2^ = 0.339. Figure 1a and b presents the changes in the MWT at the ipsilateral and contralateral sites established at the baseline; immediately after injection; and 15 min, 30 min, 45 min, 60 min, 80 min, 100 min, 120 min, 24 h, 48 h, and 7 days after the administration of iROE. For both the ipsilateral and contralateral sites, the MWT values at 15, 30, 45, and 60 min in the group administered iROE 300 mg/kg were significantly higher than those in the control, iROE 100 mg/kg, and iROE 30 mg/kg groups. The MWT values at 15, 30, 45, and 60 min in the 100 mg/kg group were significantly higher than those in the control group for both the ipsilateral and contralateral sites. The MWT at 45 min in the iROE 100 mg/kg group for the ipsilateral site and the MWT at 30 and 45 min in the iROE 100 mg/kg group for the contralateral site were significantly higher than those in the iROE 30 mg/kg group.

The AUC values showed significant differences between the groups injected at the ipsilateral and contralateral sites (F[4, 45] = 12.153, *P* < 0.001 and F[4, 45] = 7.504, *P* < 0.001). Compared with the control group, the AUC values at the ipsilateral site were significantly higher in the iROE 30 mg/kg, iROE 100 mg/kg, and iROE 300 mg/kg groups (MD 40.04; 95% CI 0.27 to 79.81; *P* = 0.048, MD 64.60; 95% CI 24.83 to 104.37; *P* < 0.001, MD 90.01; 95% CI 50.24 to 129.78; *P* < 0.001, respectively) and the AUC values at the contralateral site were significantly higher in the iROE 100 mg/kg, and iROE 300 mg/kg groups(MD 44.29; 95% CI 0.00 to 88.57; *P* = 0.050, MD 75.28; 95% CI 30.99 to 119.56; *P* < 0.001, respectively).

The LMEM showed a significant difference between the groups injected at the ipsilateral and contralateral sites (F[4, 522.85] = 33.540, *P* < 0.001 and F[4, 494.01] = 24.454, *P* < 0.001). The MWT in the iROE 10 mg/kg, iROE 30 mg/kg, iROE 100 mg/kg, and iROE 300 mg/kg groups was significantly higher than that in the control group at the ipsilateral (MD 0.15; 95% CI 0.05 to 0.25; *P* = 0.030, MD 0.23; 95% CI 0.13 to 0.33; *P* < 0.001, MD 0.40; 95% CI 0.30 to 0.50; *P* < 0.001, MD 0.54; 95% CI 0.44 to 0.64; *P* < 0.001, respectively) and contralateral sites (MD 0.10; 95% CI 0.00 to 0.21; *P* = 0.049, MD 0.28; 95% CI 0.17 to 0.38; *P* < 0.001, MD 0.44; 95% CI 0.34 to 0.54; *P* < 0.001, respectively).

### Elucidation of the mechanism mediating iROE-induced analgesia

**Fig. 2:**
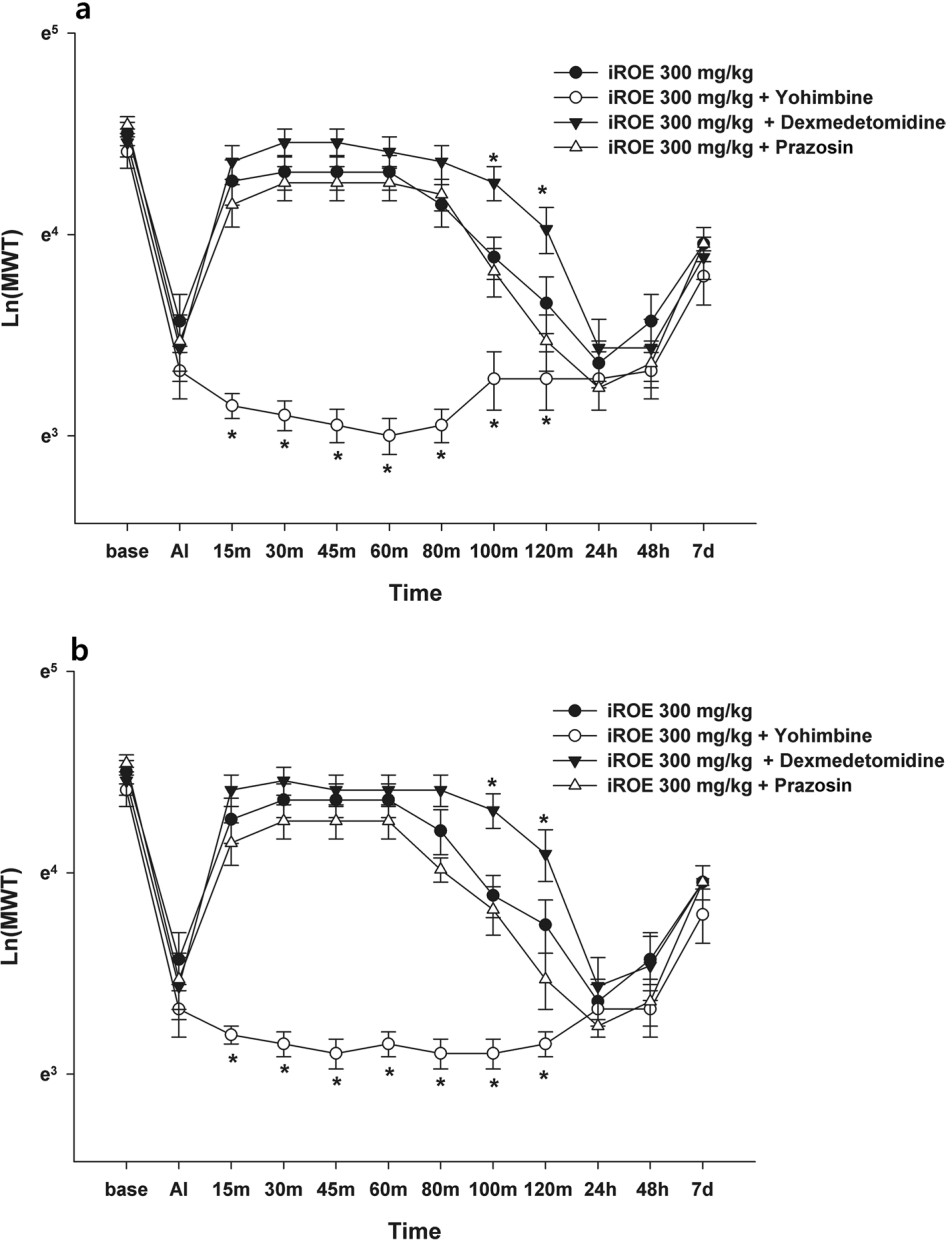
Antinociceptive mechanism of immature *Rubus occidentalis* extract (iROE) together with yohimbine, prazosin, or dexmedetomidine. **a** ipsilateral site, **b** contralateral site. AI: immediately after injection. For statistical analysis, one-way Wilk’s lambda multivariate analysis of variance followed by post hoc univariate ANOVA with the Bonferroni correction and linear mixed-effects model was used. *P < 0.05 compared with the iROE 300 mg/kg group

**Fig. 3:**
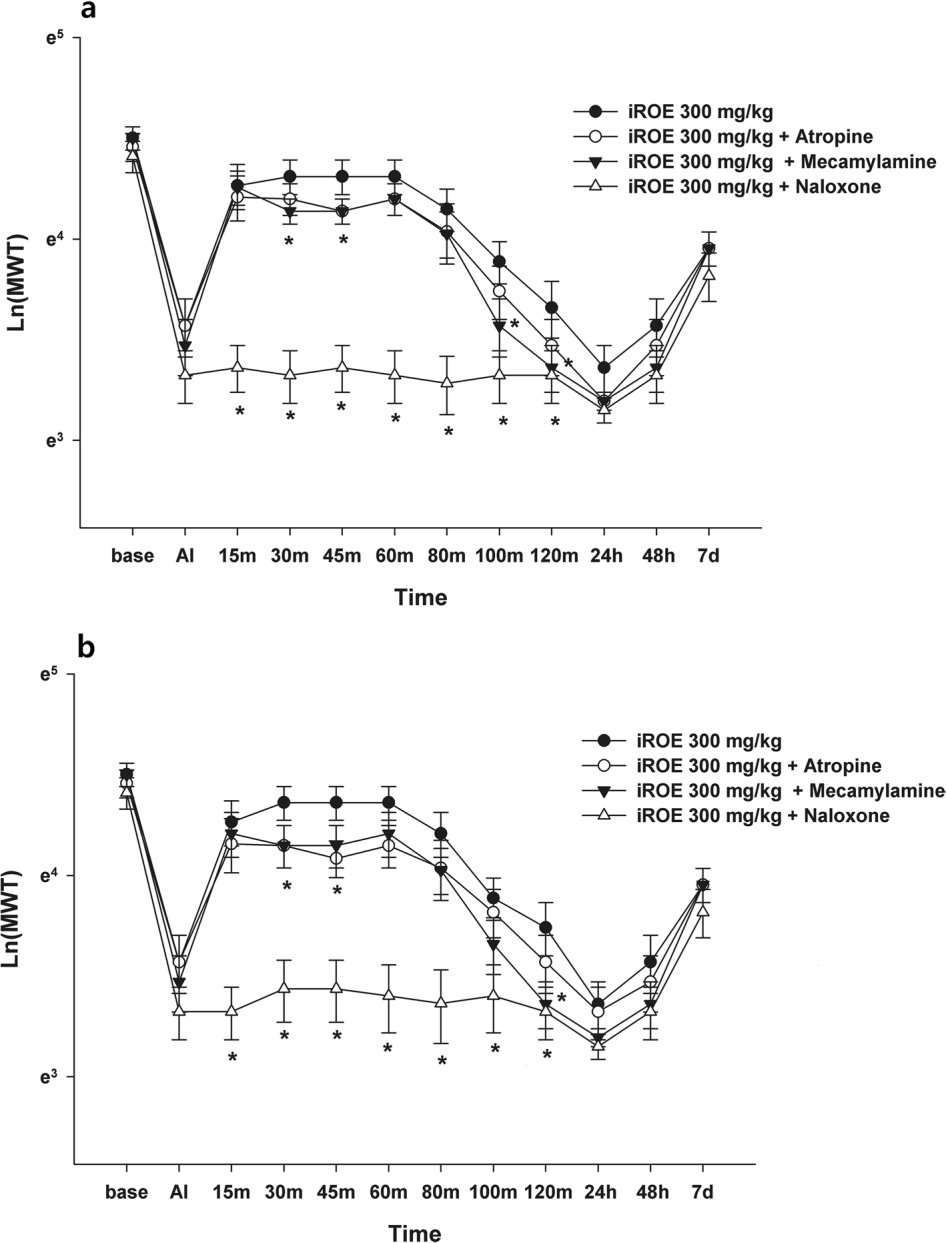
Antinociceptive mechanism of immature *Rubus occidentalis* extract (iROE) together with atropine, mecamylamine, or naloxone. **a** Ipsilateral site, **b** Contralateral site. AI: immediately after injection. For statistical analysis, one-way Wilk’s lambda multivariate analysis of variance followed by post hoc univariate ANOVA with the Bonferroni correction and linear mixed-effects model was used. *P < 0.05 compared with the iROE 300 mg/kg group

The results of MANOVA at the ipsilateral and contralateral sites showed a statistically significant difference between the groups (F(72, 288.72) = 2.906, *P* < 0.001: Wilk’s lambda = 0.052, partial η^2^ = 0.390 and F(72, 288.72) = 1.863, *P* < 0.001: Wilk’s lambda = 0.125, partial η^2^ = 0.293). For both the ipsilateral and contralateral sites, the MWT values significantly decreased at 15, 30, 45, 60, 80, 100, and 120 min in the yohimbine group and increased at 100 and 120 min in the dexmedetomidine group (Fig. 2a and b). The MWT values significantly decreased from at 15, 30, 45, 60, 80, 100, and 120 min in the naloxone group for both the ipsilateral and contralateral sites and, in the mecamylamine group, decreased at 30, 45, 100, and 120 min at the ipsilateral site and at 30, 45, and 120 min at the contralateral site (Fig. 3a and b). The LMEM showed a significant difference between the groups injected at the ipsilateral and contralateral sites (F[6, 773.90] = 82.409, *P* < 0.001 and F[6, 786.48] = 59.483, *P* < 0.001).

Compared with the MWT value for the iROE 300 mg/kg group, the MWT values were lower in the yohimbine, atropine, mecamylamine, and naloxone groups for both the ipsilateral (MD -0.70; 95% CI − 0.79 to − 0.61; *P* < 0.001, MD -0.10; 95% CI − 0.20 to − 0.01; *P* = 0.033, MD -0.12; 95% CI − 0.22 to − 0.03; *P* = 0.011, MD -0.58; 95% CI − 0.67 to − 0.49; *P* < 0.001, respectively) and contralateral sites (MD -0.61; 95% CI − 0.71 to − 0.52; *P* < 0.001, MD -0.13; 95% CI − 0.22 to − 0.03; *P* = 0.010, MD -0.15; 95% CI − 0.25 to − 0.05; *P* = 0.002, MD -0.53; 95% CI − 0.62 to − 0.43; *P* < 0.001, respectively). The MWT in the dexmedetomidine group at the ipsilateral site was higher than that in the iROE 300 mg/kg group (MD 0.10; 95% CI 0.01 to 0.20; *P* = 0.030). There was no evidence of a significant difference in the prazosin group for both the ipsilateral and contralateral sites (MD -0.06; 95% CI − 0.15 to 0.04; *P* = 0.240 and MD -0.09; 95% CI − 0.19 to 0.00; *P* = 0.061) and in the dexmedetomidine group for the contralateral site (MD 0.09; 95% CI − 0.00 to 0.19; *P* = 0.062).

The AUC values showed significant differences between the groups injected at the ipsilateral and contralateral sites (F[6, 63] = 59.958, *P* < 0.001 and F[6, 63] = 30.788, *P* < 0.001). Compared with the MWT value for the iROE 300 mg/kg group, the AUC values at the ipsilateral site were significantly lower in naloxone and yohimbine group (MD -85.61; 95% CI − 112.10 to − 59.12; *P* < 0.001, MD -105.36; 95% CI − 131.85 to − 78.87; *P* < 0.001, respectively) and the AUC values at the contralateral site were significantly lower in naloxone and yohimbine group (MD -85.98; 95% CI − 123.37 to − 50.59; *P* < 0.001, MD -107.20; 95% CI − 143.59 to − 70.81; *P* < 0.001, respectively).

### Comparison of the antinociceptive effect of iROE and mROE: Mature-immature comparison test

**Fig. 4:**
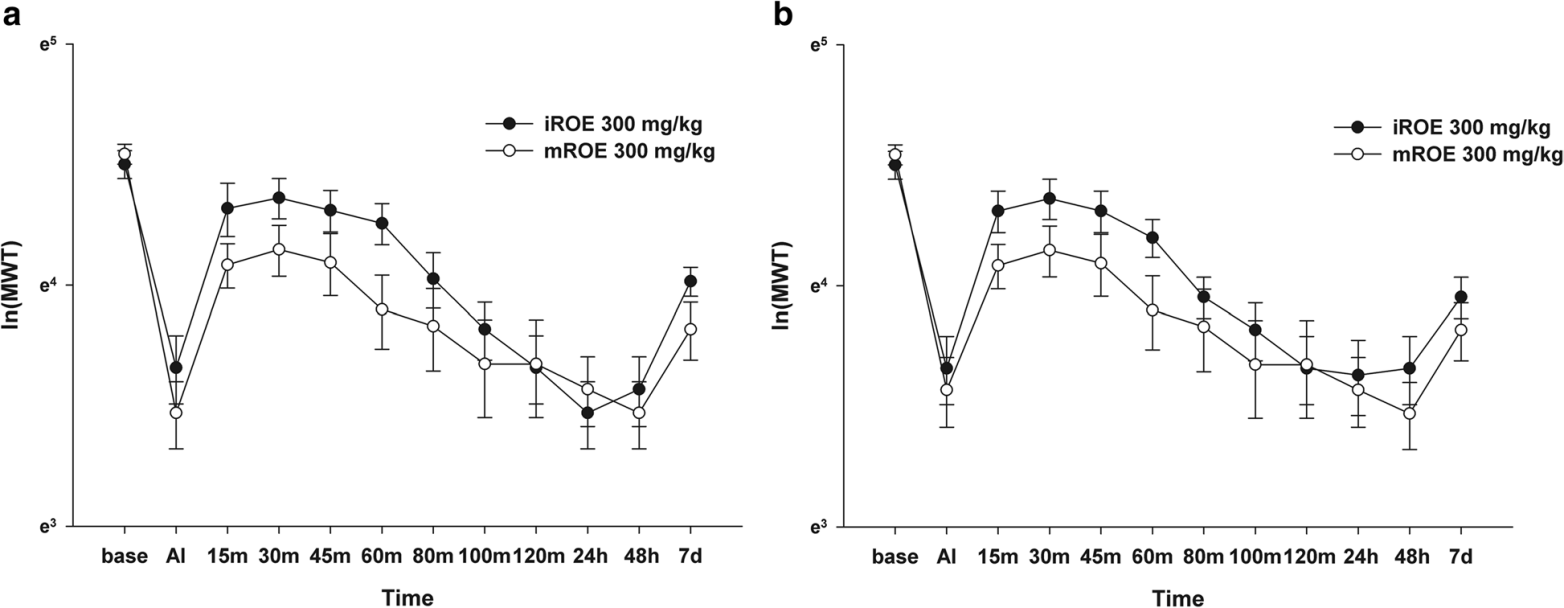
Antinociceptive effect of immature *Rubus occidentalis* extract (iROE) versus mature *R. occidentalis* extract (mROE). **a** Ipsilateral site, **b** Contralateral site. AI: immediately after injection. For statistical analysis, one-way Wilk’s lambda multivariate analysis of variance and linear mixed-effects model was used

There was no evidence of a significant difference between the iROE 300 mg/kg group and the mROE 300 mg/kg group according to the MANOVA results for the ipsilateral and contralateral sites (F[12.0,7.0] = 1.808, *P* = 0.221, partial η^2^ = 0.756 and F[12.0,7.0] = 0.850, *P* = 0.616, partial η^2^ = 0.593). The AUC values showed no evidence of significant differences between the groups injected at the ipsilateral and contralateral sites (MD 26.11; 95% CI − 5.56 to 57.77; *P* = 0.100 and MD 24.44; 95% CI − 6.15 to 55.02; *P* = 0.099, respectively). However, the LMEM showed a significant difference between the iROE 300 mg/kg and mROE 300 mg/kg groups for both the ipsilateral and contralateral sites (F[1, 163.33] = 8.211, *P* = 0.005, MD 0.13; 95% CI 0.04 to 0.22 F[1, 164.17] = 9.198, *P* = 0.003 and MD 0.14; 95% CI 0.05 to 0.23) (Fig. 4a and b).

### Comparison of the antinociceptive effect of iROE versus the positive control

**Fig. 5:**
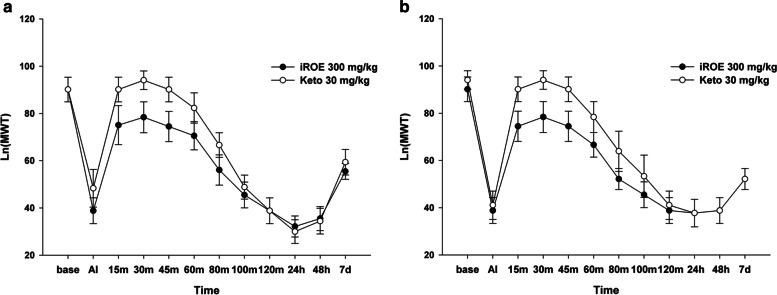
Antinociceptive effect of immature *Rubus occidentalis* (iROE) versus 30 mg/kg of ketorolac (Keto). AI: immediately after injection. For statistical analysis, one-way Wilk’s lambda multivariate analysis of variance and linear mixed-effects model was used

There was no significant difference between the iROE 300 mg/kg and positive-control groups according to the MANOVA results for the ipsilateral and contralateral sites (F[12.0,7.0] = 0.784, *P* = 0.661, partial η^2^ = 0.573 and F[12.0,7.0] = 0.474, *P* = 0.878, partial η^2^ = 0.448). However, the LMEM showed a significant difference between the iROE 300 mg/kg and positive-control groups for the ipsilateral and contralateral sites (F[1, 183.08] = 4.178, *P* = 0.043, MD 0.08; 95% CI 0.00 to 0.16 and F[1, 154.17] = 20.55, *P* = 0.001, MD 0.12; 95% CI 0.05 to 0.20) (Fig. 5a and b). The AUC values showed no evidence of significant differences between the groups injected at the ipsilateral and contralateral sites (MD 16.63; 95% CI − 3.94 to 37.20; *P* = 0.107 and MD 15.74; 95% CI − 5.80 to 37.27; *P* = 0.142, respectively). There was no evidence of a significant difference between the iROE 300 mg/kg and positive-control groups, according to the MANOVA results, for the contralateral site (F[12.0,7.0] = 0.474, *P* = 0.878, partial η^2^ = 0.448). However, the LMEM showed a significant difference between the iROE 300 mg/kg and positive-control groups.

### Assessment of motor impairment

Compared with the control, iROE 300 mg/kg and mROE 300 mg/kg showed no significant effect on motor performance, as measured by the Rota-rod test, 30 min after the intraperitoneal injection of ROE (*P* = 0.533). The time points at which the rats fell during the Rota-rod test were 107.83 ± 11.65, 104.33 ± 10.74, and 100.67 ± 10.07 in the control, iROE 300 mg/kg, and mROE 300 mg/kg groups, respectively.

## Discussion

This study is the first, to our knowledge, to present the antinociceptive effect of the intraperitoneal administration of immature *R. occidentalis* in rats with hyperalgesia induced by repeated intramuscular injection of acidic saline. Our findings showed that iROE exhibited an antinociceptive effect in a dose-dependent manner. This analgesic property of iROE was significantly agonised by dexmedetomidine and antagonised by naloxone, mecamylamine, and yohimbine, which indicates the involvement of the α_2_-adrenergic, cholinergic, and opioid receptors. Moreover, iROE presented a superior antinociceptive potential to mROE.

Since we observed that mROE exhibited an analgesic effect on chronic muscle pain in previous study [7], we would like to perform the study regarding iROE and compare between mROE and iROE in current study utilizing dose of 300 mg/kg with greatest analgesic effect. Although MANOVA revealed no evidence of a significant difference between the immature and mature groups, LMEM revealed a significant difference. We were unable to draw a firm conclusion about our result because MANOVA was used during the sample size calculation stage. However, based on the LMEM results, we can propose that immature *R. occidentalis* has a higher potential for analgesic effect than mature fruit. Besides, several studies on immature fruit provided support for our LMEM-based results. This may be the first and valuable evidence for the beneficial effect of immature black raspberry on chronic pain management.

The content of ellagic acid and anthocyanin, major components of ROE, depends on the ripening of the fruit [5, 17]. Several studies have reported the different capacity of bioactive components in fruits including *R. occidentalis*. A study investigating the bioactive and pharmacokinetic characterisation of immature *R. occidentalis* reported that the immature fruit produced a high content of ellagic acid and vitamin C, presenting superior antioxidant activities [6]. Similar results have been observed for other *Rubus* species [6, 18]. Specifically, ellagic acid, a bioactive component in *Rubus* species, was significantly higher in immature *R. coreanus* fruit than in mature fruit. Taghi et al. found that ellagic acid had central and peripheral antinociceptive effect in experimental pain model [19], which supports the analgesic effect of immature *R. occidentalis* based on our findings. In addition, *R. coreanus* fruit have potential applications owing to their antioxidative effects mediated via the scavenging of reactive oxygen species [20]. Therefore, it is suggested that components with the observed antioxidative activity are degraded during the ripening process. This theory can be supported by our results of the comparison between iROE and mROE: iROE showed the potential of superior analgesic effect to mROE. The antioxidative property of *R. occidentalis* is worthy of consideration for the management of chronic pain. Oxidative stress refers to a condition that involves a disturbance in the balance between the production of oxidants and the reactivity of antioxidants; this balance is normally well-controlled [21]. There is strong evidence suggesting that oxidative stress is present in the skeletal muscle of patients with chronic diseases. Besides, since oxidative stress is intensively involved in the pathology of chronic fatigue, its levels are increased in chronic fatigue syndrome and are associated with clinical symptoms such as chronic musculoskeletal pain [22]. There is a causal link between oxidative stress and the pathogenesis of disease, and oxidative stress is now considered a major contributor to the development of numerous chronic diseases with pain [21]. Thus, the antioxidative effect of immature *R. occidentalis* can be utilised in chronic pain control, which supports its potential as an effective dietary complement for the management of chronic pain.

Our results suggested that the α_2_-adrenergic and nicotinic cholinergic receptors were involved in the mechanism of antinociception. The involvement of the α_2_-adrenergic and nicotinic cholinergic receptors in the effect of iROE, as observed in the current study, was in agreement with the results of the previous study. Analgesic property of dexmedetomidine associated with α_2_ adrenoceptor is now well-established in perioperative pain management. It is useful for multimodal analgesic strategy in chronic pain patients for whom opioid anlagesics are ineffective [23]. Nicotinic acetylcholine receptors can be a therapeutic target for the chronic pain control [24]. Desensitization of the α4β2* nicotinic acetylcholine receptor may contribute to pain management efficacy and may be a mechanism for nicotinic agonist-mediated analgesic effects [25]. Hence, immature *R. occidentalis* may be a promising candidate for multimodal analgesic therapy in patient suffering from chronic pain. It is interesting that current finding that opioid receptors were linked to the analgesic effect of iROE was contradictory to previous study’s finding. This is a clear point of difference between mROE and iROE, which makes therapeutic approach specifically using iROE. Given the need to replace of opioid use in clinical field, iROE appears to be more beneficial than mROE in trems of a multimodal analgesic approach [18]. Non-steroidal anti-inflammatory drugs, such as ketorolac, which is widely administered intravenously and intramuscularly, have the potential to replace opioids in pain management. We discovered that iROE of 300 mg/kg showed an expected analgesic effect compared to ketorolac, implying the potential clinical availability of immature *R. occidentalis*.

The current study has some limitations. First, the chronic pain model used in our study does not cause inflammation. Since there is strong evidence of the beneficial effect of *R. occidentalis* against inflammation, it would be valuable to obtain additional evidence by conducting further studies using the chronic inflammatory pain model. Second, despite the fact that we discussed the analgesic property of immature *R. occidentalis* in relation to its component, we did not conduct experiments at the molecular or cellular level. Further research that can provide evidence in this regard will be required. Third, although we compared the analgesic activity between iROE and mROE, there was no further comparison between the two in terms of several beneficial properties such as an antiproliferative or anti-inflammatory effect. Future experiments based on an extended study design with a larger sample size may provide valuable evidence regarding the difference between immature and mature *R. occidentalis*. Finally, the current study administered ROE and test drugs intraperitoneally, which raises concern that this route may not be suitable for the experimental study of black raspberry. However, the intraperitoneal route is simple to set up, quick, and has a low stress impact on laboratory rodents [26], making it useful in pain research. Based on our findings from previous and current studies, future research that implements ROE via oral administration should take clinical applicability into account, which is supported by a study reporting the role of black raspberry in cancer and variations in fruit maturity stage [3].

Nonetheless, this study is valuable as an experimental study of *R. occidentalis* because it is based on a well-established and meticulously designed research protocol from our previous studies [7, 27]. We have made several attempts to investigate the effects of *R.occidentalis* in various pain models in greater depth. While previous studies presented evidence for ripe fruits, this study focused on presenting evidence for immature fruits as well as deriving new evidence by comparing immature and mature fruits. Our findings with *R. occidentalis* in both the acute and chronic pain models are expected to provide very useful evidence for clinical availability of *R. occidentalis*. This kind of research is characterizing the bioactivities of plant products and expanding their use in human healthcare.

## Conclusions

Immature *R. occidentalis* showed antinociceptive activity against acid-induced chronic muscle pain. Its action may be mediated by the α_2_-adrenergic, nicotinic cholinergic, and opioid receptors and iROE displayed a superior antinociceptive tendency to mature ROE.

## Supplementary Information

**Additional file 1.**

## Data Availability

The datasets used and analysed during the current study are available from the corresponding author upon reasonable request.

## Abbreviations

ANOVA: Analysis of variance
ARRIVE: Animal Research: Reporting In Vivo Experiments
IP: Intraperitoneal
iROE: Immature *Rubus occidentalis* extract
LMEM: Linear mixed-effects model
MANOVA: Multivariate analysis of variance
MD: Mean difference
mROE: Mature *Rubus occidentalis* extract
MWT: Mechanical withdrawal threshold
ROE: *Rubus occidentalis* extract
Rpm: Rotations per minute
