# Supplementary material for: Effectiveness of maturity of Rubus occidentalis on hyperalgesia induced by acidic saline injection in rats
Source: BMC Complement Med Ther. 2022 Jan 11;22:12. doi: 10.1186/s12906-021-03491-z (PMC8751266; doi:10.1186/s12906-021-03491-z)
Supplement: Supplementary file 1 — Additional file 1. [file 12906_2021_3491_MOESM1_ESM.docx]

**Statistical Analysis**

The primary aim of Experiment 1 was to estimate the differences in the MWT using von Frey filaments between the control, iROE 10 mg/kg, iROE 30 mg/kg, iROE 100 mg/kg, and iROE 300 mg/kg groups. To estimate the required group size for Experiment 1, the MWT of the control group from a previous study was considered [7]. As the MWT of the control group did not pass the Shapiro-Wilk test, the data were transformed using natural logs. The averages of the natural log-transformed MWT at baseline; immediately after injection; 15, 30, 45, 60, 80, 100, and 120 min post-injection; 24 and 48 h post-injection; and 7 days post-injection were 4.52, 1.93, 1.93, 1.93, 1.97, 1.97, 1.97, 1.97, 1.97, 1.97, 2.59, and 2.80 ln (mN), respectively. The standard deviations of the natural log-transformed MWT ranged from 0.01 to 0.64 ln(mN), and the autocorrelation between adjacent measurements on the same rat was 0.6. For our power calculations, we assumed that a first-order autocorrelation adequately represented the autocorrelation pattern. To compare the between-group difference, we planned to use the Geisser-Greenhouse Corrected F test for the repeated measures analysis of variance (ANOVA). We aimed to detect a 20 %, 30 %, 40 %, and 50 % increase in the MWT in the iROE 10 mg/kg, iROE 30 mg/kg, iROE 100 mg/kg, and iROE 300 mg/kg groups compared with that in group C. Consequently, the standard deviation was 0.20, and the actual effect standard deviation was 0.14; therefore, the effect size was 0.70. With an α of 0.05 and a power of 80 %, we needed 8 rats per group. Considering a 20 % follow-up loss, we allocated 10 rats to each group.

The Shapiro-Wilk test was used to test the normality of the variables. As the MWT did not pass the Shapiro-Wilk test, natural log-transformation was performed for the MWT, and the natural log-transformed MWT passed the Shapiro-Wilk test. We therefore assumed that the normal distribution assumption for the parametric test was not violated and decided to apply repeated measures ANOVA**.** Because Mauchly’s sphericity test indicated that the assumption of sphericity had been violated, we used one-way Wilk’s lambda multivariate analysis of variance (MANOVA): each group as an independent factor and the MWT at each time point as dependent variables. To compare the MWT at each time point, univariate ANOVA or t-tests with the Bonferroni correction (α = 0.05/12 = 0.0042) were used. When the homoscedasticity requirement using Levene’s test for the homogeneity of variances was not met in the ANOVA, we used Welch’s corrected ANOVA. Tukey’s or Tamhane’s T2 post-hoc test was used when ANOVA or Welch’s corrected ANOVA was significant to identify the groups with statistically significant mean differences (MD). In addition, the between-group difference for the MWT was analysed using the linear mixed-effects model (LMEM), which was obtained using time points and groups as independent fixed factors and individual rats as random effects.
